# Leakage reduction for the Siemens ModuLeaf

**DOI:** 10.1120/jacmp.v10i2.2894

**Published:** 2009-04-22

**Authors:** R. Alfredo Siochi

**Affiliations:** ^1^ Department of Radiation Oncology University of Iowa Hospitals and Clinics Iowa City Iowa U.S.A.

**Keywords:** MultiLeaf Collimator, commissioning, treatment planning system

## Abstract

The ModuLeaf, an add‐on miniature multileaf collimator (MMLC) for the Siemens ONCOR linear accelerator, provides high resolution field shaping with a maximum interleaf leakage dose of 1.50% at 6 MV. However, beyond the maximum treatment field size, the distribution of leakage and scatter along the y‐axis is different from that of the x‐axis, with maximum leakage values of 1.53% and 0.39%, respectively. Such differences cannot be modeled in the Pinnacle treatment planning system. Also, within the 10 cm×12 cm treatment region, leakage from the crack between closed leaf ends was 3.76%. To resolve these issues, gaps in the ModuLeaf frame were filled with lead sheets, and the Siemens MLC was operated in MLC mode (rather than bank mode). As a result, a rectangle of 10.4 cm×11 cm was formed with the MLC leaves closed behind the Y jaws, whose opening was 10.4 cm. This significantly reduced the difference between the leakage patterns in the x and y directions, with maximum leakage doses of 0.43% outside the treatment region and 1.67% near the crack between abutting ModuLeaf leaves. The modification also reduced the mean square error between Pinnacle profiles and measured profiles in the tail region.

PACS number: 87.56.jk

## I. INTRODUCTION

The ModuLeaf (Siemens Medical Systems, Concord, CA) is an add‐on miniature multileaf collimator (MMLC) with 40 leaf pairs, a projected leaf width of 2.5 mm at an SSD of 100 cm, a maximum field size of 12 cm×10 cm on a Siemens ONCOR accelerator, and a leaf positioning tolerance of 0.5 mm.^(1)^ It provides highly conformal fields with improved critical structure shielding.

In order to benefit from the improved precision and accuracy, the calculation of dose to avoidance structures must be reliable. For example, in a majority of pituitary adenoma cases, the optic chiasm is within 1 mm of the tumor.^(2)^ If a dosimetrist is able to shield the chiasm behind a 2.5 mm leaf, the chiasm will still receive leakage and scatter dose contributions that must be calculated accurately. Hence, beam models for extra‐focal radiation should fit profiles measured in cross‐plane (x‐axis) and in‐plane (y‐axis) directions. Our attempts at creating Pinnacle 6 MV photon beam models led to compromises since the measured profiles behave differently starting around 5 cm from isocenter, with the y profiles consistently higher than the x profiles. It was apparent that there was extra leakage through the ModuLeaf frame along the y‐axis that was not present on the x‐axis. The calculated profiles matched the measured data on the x‐axis at all points for both small and large fields. For the y‐axis, the calculated profiles matched the measured data within the interior of the field (as determined by the area with doses greater than 50% of the central axis dose); however, outside the field, calculated values were lower than the measured ones, especially for larger fields. This region is important for situations that require a large number of monitor units (e.g. IMRT, Stereotactic Radiosurgery).

Since we could not model the device to meet our needs, we modified it to improve its leakage properties. The need for modification was not anticipated, since the ModuLeaf has been in use at other clinics. However, modifications are not unusual over the lifetime of a device. Different models of MMLCs have been through design changes to reduce the leakage between the sides of adjacent leaves (interleaf leakage), through the center of a leaf (leaf transmission), and at the abutment between the tips of leaves on opposite banks (leaf‐end leakage). The mean leaf transmission and interleaf leakage values of 0.93% and 1.18%, respectively, represent the lowest values that have been measured at 6 MV for the BrainLAB m3‐MMLC.^(3)^ This is an improvement over the corresponding values previously measured by Cosgrove et al.^(4)^ (1.90% and 2.80%, respectively). Other authors reported values between these two extremes.^(^ ^5^ ^–^ ^7^ ^)^ Cosgrove et al.^(4)^ also reduced the mean leaf‐end leakage from 15.00% to 4.50% by moving the abutment at central axis to 4.5 cm off‐axis, while Agazaryan et al.^(5)^ later measured a lower mean leaf‐end leakage of 3.56%. To reduce this leaf‐end leakage, Belec et al.^(6)^ moved the abutment to 5 cm off‐axis and used linac jaw settings of 9.8 cm×9.8 cm so that the abutment is shielded by a jaw. For the Wellhofer MMLC, a leaf‐end leakage of 2.90% was reduced to 1.20% after the leaf alignment was corrected.^(8)^

We also had to modify the operating configuration of the ModuLeaf for a number of reasons. Our modified configuration has the linac jaws fixed to a 10.4 cm×10.4 cm field. Meeks et al.^(8)^ noted improved output factor stability using fixed jaws at 9 cm×9 cm, while Wang et al.^(9)^ described technical issues for the Radionics MMLC that required the use of fixed jaws at 10 cm×10 cm for IMRT delivery on a Siemens' Primus linac. Hartmann and Föhlisch used a fixed field of 12 cm×10 cm on their ModuLeaf, but they did not provide a rationale.^(1)^

None of the reviewed literature reported unusual leakage patterns like the one we observed along the y‐axis. Nor did they report any problems with beam modeling in their treatment planning system. This was also the case for other reports on the ModuLeaf, and may be due to its use with other linear accelerators,^(^ ^10^ ^–^ ^11^ ^)^ leakage films that were limited to 10 cm×10 cm,^(1)^ ^,^ ^(12)^ different mounting methods,^(1)^ ^,^ ^(12)^ or other planning systems.^(12)^ The unusual leakage pattern we observed cost us many man‐hours of work that we could have saved if we had known its effects prior to commissioning. We hope that this technical note will reduce the effort for others who intend to implement the ModuLeaf.

## II. METHODS

**Figure 1:**
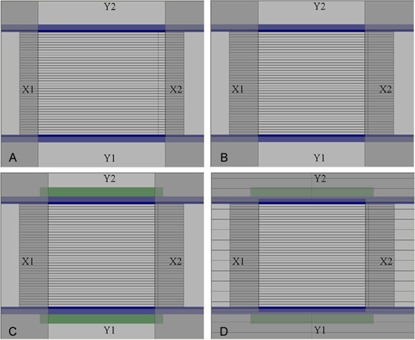
MLC and ModuLeaf configurations: (A) the field size of 12.4 cm×10.4 cm is defined by the linac X and Y jaws; (B) the modified linac X field size of 10.4 cm allows the ModuLeaf leaf abutment to hide behind the X2 jaw; (C) lead (green bars) is added beside the frame (blue bars) of the MMLC leaves; (D) the linac X jaws are operated in MLC mode so that the MLC leaves covered by the Y jaws can be closed.

Initial leakage measurements were performed with the unmodified ModuLeaf configured according to the manufacturer's recommendations, as shown in Fig. 1(A). The ModuLeaf was mounted to a Siemens ONCOR linac such that the ModuLeaf leaves were parallel to the leaves of the linac's integrated 41 leaf pair MLC (whose leaf width projected to isocenter is 1 cm). The linac's MLC operates in bank mode, where all the leaves on a side are positioned to the same x coordinate. The MLC then behaves as a pair of X jaws, which were opened to a symmetric field size of 12.4 cm. The symmetric field size for the linac's Y jaws was 10.4 cm. For all tests in this article, the ModuLeaf leaves were closed at x=5.5 cm away from isocenter. The linac collimator and gantry angles were zero for all measurements. For this beam geometry, the linac's Y jaws move along the in‐plane direction while the leaves of the linac's MLC and the leaves of the ModuLeaf move along the cross‐plane direction.

**Figure 2:**
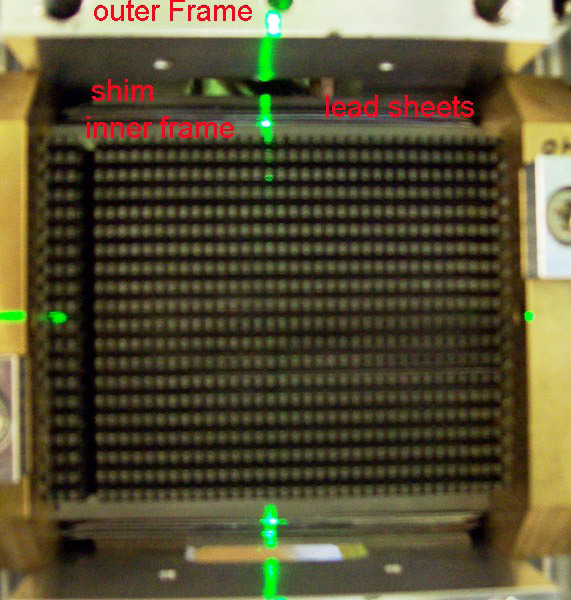
Close‐up photo of the modified ModuLeaf with lead sheets inserted in the gap between the inner and outer frames. Stainless steel shims are placed between the outer frame and the lead sheets to hold them in place. Note: X2 is on the left.

Figures 1(B), 1(C), and 1(D) show the other configurations that were explored in order to reduce leakage. Figure 1(B) is similar to Fig. 1(A), except that the MLC X jaws are coned down to 10.4 cm. This is just enough to hide the crack between the tips of opposing ModuLeaf leaves behind the X2 jaw. This crack is exposed in Fig. 1(A). Figure 1(C) is similar to Fig. 1(B), except that we have now added lead to the voids in the ModuLeaf frame, as shown in Fig. 2. Figure 1(D) is similar to Fig. 1(C), except that the linac's MLC leaves are operated in MLC mode so that the leaves shielded by the Y jaws can be closed.

The ModuLeaf was modeled in the Pinnacle planning system for the configurations in Figs. 1(A) and 1(D). Commissioning data for the Pinnacle beam models were collected using a PTW MP3 scanning water tank with a PTW 0.125 cc farmer‐type ionization chamber and a PTW 0.015 cc pinpoint ionization chamber (PTW, Freiburg, Germany).

**Figure 3:**
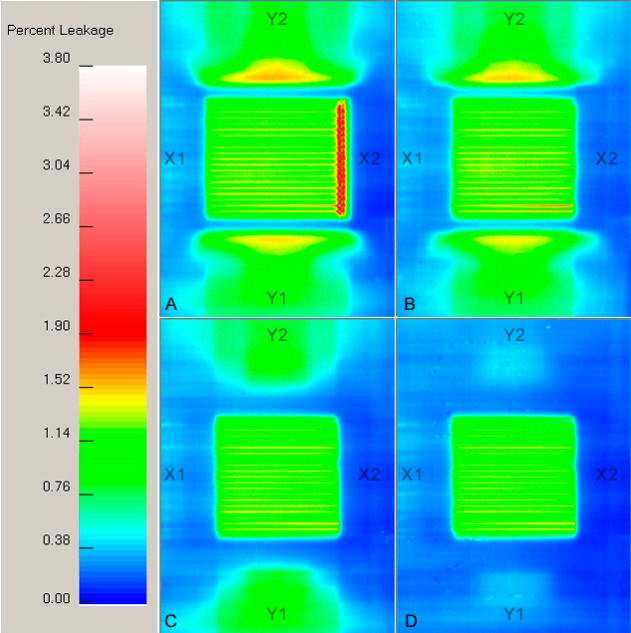
Percent leakage films corresponding to the configurations in Fig.1. The color bar on the left is the percent leakage scale. The labels X1, X2, Y1, and Y2 identify the linac's jaws in beam's eye view. X jaw opening=12.4 cm; (B) X jaw opening=10.4 cm; (C) lead is added; (D) the MLC mode is used.

Film densitometry measurements for the configurations in Figs. 1(A) to 1(D) are shown in Figs. 3(A) to 3(D). The measurements were performed using Kodak EDR‐2 film sandwiched between two solid water slabs of dimensions 40 cm×40 cm×5 cm at an SSD of 95 cm and a depth of 5 cm. The films were exposed to 3,000 MUs of 6 MV X‐rays. The developed films were scanned on a Microtek Scanmaker 9800 XL flatbed scanner with a TMA 1600 transparency media adapter (Microtek, Cerritos, CA). The doses on the leakage film are determined by logarithmic interpolation of the values in the calibration table. The table was determined using the scanned image of a calibration film with 12 square regions that have been exposed to different doses. These doses are mapped to the corresponding intensities on the scanned image. An unexposed film was also developed to determine the image intensity that corresponds to zero dose. Table 1 shows a typical digital film densitometry calibration table.

**Table 1:** Typical film image intensity to dose conversion table.

*Intensity* ^*^	*Dose, cGy*
10962	295.71
16639	256.14
21499	219.12
27877	177.00
32615	147.77
36624	120.36
42437	88.78
46373	69.98
49963	51.32
55338	31.46
58482	22.03
61308	12.43
65259	0.00

* Maximum intensity=65535.

**Figure 4:**
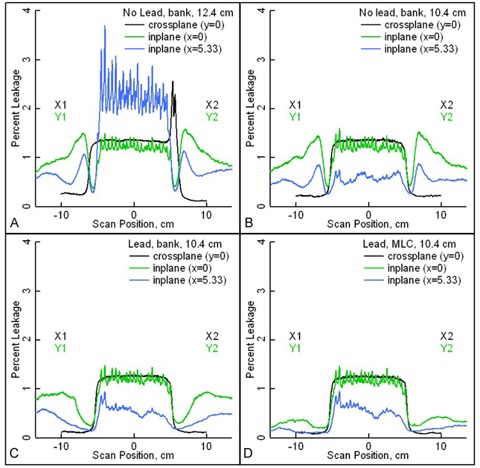
Profiles from the corresponding leakage films in Black and green curves are x and y profiles, respectively, while blue curves are parallel to the y‐axis under the ModuLeaf leaf‐end abutment. The labels X1, X2, Y1, and Y2 indicate the position of the linac jaws relative to the profiles.

Figures 4(A) to 4(D) show the film profiles along the x‐ and y‐axes through isocenter (black and green curves, respectively), as well as the profile along the y‐axis under the “crack” (blue curve), near the ModuLeaf closed leaf position (at x=5.33 cm). Although the leaves close off‐axis at a nominal 5.5 cm, the actual hot spot is around 5.33 cm. The films and their corresponding profiles were all normalized to 2825 cGy. This is the dose that the film, at a depth of 5 cm, would have received at isocenter if the ModuLeaf were opened to 10 cm×10 cm. Hence Figs. 3 and 4 represent percent leakage values.

The linac X jaw (MLC) positions in Figs. 1(B) to 1(D) were determined as a compromise between leakage from the crack (Fig. 1(A) and ModuLeaf field size. The X jaw position recommended by the manufacturer is 6.2 cm (i.e. a field size of 12.4 cm, seen in Fig. 1(A). If the MLC leaves that form the X jaw stray from their calibration by less than 0.2 cm, they will not invade the maximum ModuLeaf X field size of 12 cm. Hence, the field edge at 6 cm is defined by the ModuLeaf leaves and not by the linac's MLC. We have observed in our clinic that the linac's MLC leaves can vary from their calibrated position by as much as 2 mm, in agreement with the manufacturer's recommended margin. However, by allowing the ModuLeaf leaves to move up to 6 cm from isocenter, cracks between the ModuLeaf's closed leaf tips will not be able to hide behind the linac's MLC. The closed leaf position is at 5.5 cm; hence the X jaw position must be less than 5.5 cm. Since we need a margin of 0.2 cm to allow for linac MLC calibration drift, the X jaw position must be less than 5.3 cm. Table 2 shows the maximum leakage observed as a function of symmetric X field size, without any lead inserted in the ModuLeaf. This was determined from additional leakage films that were taken in the same manner as those in Figs. 3(A) and 3(B), but with the X field sizes listed in Table 2. If we use a jaw position of 5.2 cm, and we account for variability in the linac's MLC calibration, then the linac's X jaw could move between 5.0 cm and 5.4 cm. This corresponds to a linac X field size between 10.0 cm and 10.8 cm. As shown in Table 2, this means that we have a leakage range of 1.67% to 2.24%. This also means that the maximum ModuLeaf X field size we could use without the linac's MLC invading the field is 10 cm. We debated using an X field size of 10 cm, which would mean a range of 9.6 cm to 10.4 cm when the linac MLC calibration is accounted for. This would limit the leakage to less than 1.80%, but it would also limit the ModuLeaf X field size to 9.6 cm. We opted to keep a maximum ModuLeaf X field size of 10 cm for simplicity, since this is also the maximum field size in the Y direction. Moreover, the primary mode for the linac MLC calibration drift is for the MLC leaves to move towards isocenter; hence, the X field size is more likely to shrink, reducing the likelihood of exceeding a leakage of 1.80%.

**Table 2:** Maximum leakage as a function of X jaw field size (no lead).

*X Field, cm*	*Max Leakage, %*
9.0	1.62
9.6	1.66
10.0	1.67
10.2	1.73
10.4	1.79
10.6	1.86
10.8	2.24
11.0	3.08
12.4	3.76

Some ModuLeaf users^(^ ^10^ ^–^ ^12^ ^)^ move the X and Y jaws of the linac to form a tight bounding rectangle to track the ModuLeaf field shape, potentially decreasing the leakage even further. For small field sizes, a drift of 2 mm in the linac's MLC calibration would cause problems for output factor stability. This would also complicate IMRT treatment planning, since the dosimetrist would have to position the X and Y jaws for each segment, with a 2 mm margin to the shape formed by the ModuLeaf. Furthermore, it could lead to problems with the model of the toe and the tail near the penumbra region of the beam profiles and cause IMRT dose distribution errors.^(13)^ Finally, it could slow down the delivery of IMRT treatments. Hence, implementing the ModuLeaf in this mode requires much more careful study. We decided to gain experience with the system with the X and Y jaws forming a fixed 10.4 cm×10.4 cm square for all ModuLeaf shapes, prior to exploring the use of tracking jaws. As previously noted, other MMLC users have successfully employed fixed X and Y jaws.^(1)^ ^,^ ^(8)^ ^,^ ^(9)^

The placement of lead resulting in the configurations of Figs. 1(C) and 1(D) was facilitated by the use of electronic portal images (EPI) of various test conditions. All EPIs were positioned at a source‐to‐image‐distance of 146 cm and were exposed to 300 MU of 6 MV photons. By analyzing the EPIs, we traced the leakage to the gaps next to the ModuLeaf leaf banks. Each of the gaps between the outer and inner frames of the ModuLeaf was filled with 12 lead sheets measuring 8 cm×8 cm×0.5 mm. The lead sheets were held in place with stainless steel shims positioned against the outer frame of the gap, as shown in Fig. 2.

**Figure 5:**
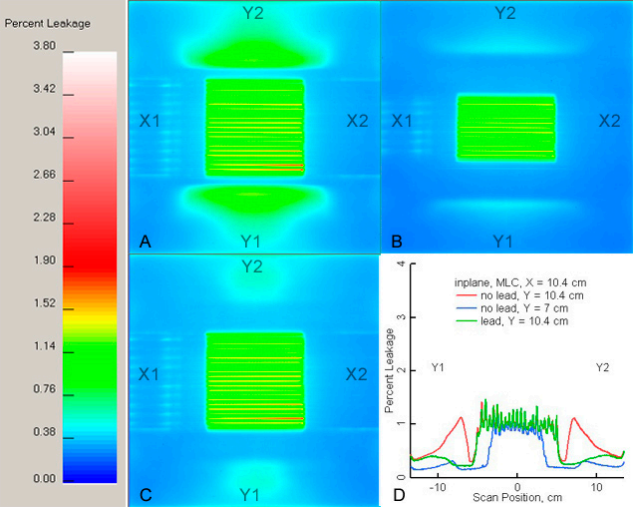
Percent leakage estimated from electronic portal images. For all images, the MLC leaves shielded by the Y jaws are closed. In (A) and (B), no lead was placed in the ModuLeaf frame. (A) both linac X and Y field sizes are 10.4 cm; (B) the linac Y jaws are coned down to a field size of 7 cm; (C) lead is added to the configuration in (A); (D) profiles along the y‐axis for the EPIs: (A) red, (B) blue, and (C) green.

Prior to placing the lead, we explored the possibility of operating the linac's MLC in MLC mode (rather than as X jaws in bank mode) so that all MLC leaves were closed except for the central 11 leaves that formed a 10.4 cm opening. We also explored the possibility of reducing the ModuLeaf field size along the y‐axis by moving the Y jaws into the field. Figs. 5(A) to 5(C) all use the MLC mode. Figure 5(B) includes the reduction of the Y field size to 7 cm, while Fig. 5(C) shows the addition of lead (same configuration as Fig. 3(D). We did not take films for these configurations and, rather than removing the lead from the ModuLeaf (which would take it out of clinical use), we decided to estimate the percent leakage from the fluence patterns in the EPIs. We had also taken an EPI (not shown) that corresponds to the film in Fig. 3(A). The percent leakage values from the film were plotted against the corresponding gray scale pixel values from that EPI. The best fit to the plot was a straight line with a correlation coefficient of 0.996: PL=0.007257*PV‐0.13, where PL=percent leakage and PV=Pixel Value. We applied this conversion to the pixel values of the EPIs to obtain the estimated percent leakage in Fig. 5.

## III. RESULTS AND DISCUSSIONS

Figures 4(A) and 4(B) indicate the importance of hiding the ModuLeaf closed leaf tips behind the linac's X2 jaw. The blue curve in these figures is the leakage along a profile through the abutment. The maximum leakage in Fig. 4(A) (and over the entire area in Fig. 3(A) is 3.76%, while in Fig. 4(B) the maximum leakage under the abutment is 0.75%, which is less than the interleaf leakage on the y‐axis (green curve). The maximum leakage over the entire area in Fig. 3(B) moves away from the abutment to some point near the edge of the linac's X2 jaw, and its value is 1.81%. Figures 3(C) and 3(D) also have a lower maximum leakage near the linac's X2 jaw, with values of 1.69% and 1.67%, respectively. It is debatable whether this difference of 0.12% to 0.14% compared to Fig. 3(B) (1.81%) is due to the lead (Fig. 3(C), the MLC mode and the lead (Fig. 3(D), the slight variations in MLC leaf positions on the X2 side, or film densitometry errors.

The leakage profiles in Figs. 4(A)) to 4(D) show that beyond the ModuLeaf treatment region (|y|>5.3 cm or |x|>5.3 cm), the y‐axis (green curve) has a higher maximum leakage than the x‐axis (black curve). However, the y‐axis maximum is reduced from 1.53% (Fig. 4(B) to 0.94% (Fig. 4(C) by adding lead. In addition to the lead, closing the leaves behind the Y jaws reduces the y‐axis maximum leakage to 0.43% (Fig 4(D)). Clearly, both modifications are needed to reduce the leakage to levels that are comparable with those of the x‐axis (a maximum of 0.39% in the region |x|>5.3 cm).

Figures 5(A) and 5(C) indicate that using the MLC mode without the lead is not as effective as combining it with the lead. The maximum y‐axis leakage in the region |y|>5.3 cm is 1.13% when the MLC mode is used (red curve, Fig. 5(D), which is higher than 0.43%, the value obtained when lead is used with the MLC mode (green curve, Figs. 4(D) and 5(D). However, if instead of adding lead one is willing to cone down the linac's Y jaws to a 7 cm opening, the maximum y‐axis leakage can be diminished to similar levels. The blue curve in Fig. 5(D) indicates a maximum leakage value of 0.32% for |y|>3.8 cm. We tried the same experiment with the Y jaws opened to a field size of 9 cm, but we could not reduce the leakage value below 0.65% for |y|>4.8 cm. By adding lead, we are able to use the full y‐axis field size of 10 cm for the ModuLeaf. Hence, we decided to use the configuration in Fig. 1(D) in clinical applications.

Comparing our clinical configuration in Fig. 4(D) to the original configuration in Fig. 4(A), the profiles indicate similar interleaf leakage and leaf transmission values. Prior to the modification, the minimum, mean, and maximum interleaf leakage values were 0.94%, 1.31%, and 1.5%, respectively. After the modification, the corresponding values were 0.86%, 1.26%, and 1.48%. While the modified leakage values are lower by 0.05%, these values are within experimental error of each other, so the modification did not affect the interleaf leakage. A similar behavior was noted for the leaf transmission values. Prior to the modification, the minimum, mean, and maximum leaf transmission values were 0.88%, 1.13%, and 1.20%, respectively. After the modification, the corresponding values were 0.83%, 1.08%, and 1.15%.

A comparison of leaf‐end leakage is clinically meaningless, since the leaf‐ends are hidden behind the X2 leaves of the MLC in Fig. 3(D). A more appropriate metric is the maximum leakage observed over the whole film. In the case of the original configuration, this also happens to be the maximum leaf‐end leakage and has a value of 3.76%. After the modification, this was reduced to 1.67%. Note that these values occur over a very small region (around 0.3 mm × 0.3 mm) of the film, and this would be blurred over several treatment beam angles and treatment fractions. For the clinical configuration, the average value of the maximum leakage in a 3 mm square centered on the hot spot was 1.36%. This is similar to the interleaf leakage values. This is a consequence of the hot spot being near the edge of the linac's X2 jaw (formed by MLC leaf tips on the X2 side) where the leakage drops off very quickly. This decreases the average value near the hot spot.

The modified MMLC also had symmetric commissioning data, with the x and y profiles for square fields being nearly identical, except in the penumbra region. This enabled us to obtain an improved fit for the Pinnacle beam model. Table 3 shows the mean square error (relative to central axis) between the Pinnacle profiles and the measured data for the original and modified ModuLeaf. The error analysis was limited to the tail region, since this is where compromises were made between the in‐plane and cross‐plane profile results for the original ModuLeaf. On average, the differences in the errors between the x‐ and y‐axes were 0.78% and 0.06% for the original and modified ModuLeaf, respectively. This indicates a higher similarity between the tails of the × and y profiles for the modified ModuLeaf. Also, the averages over all errors in Table 3 were 0.79% and 0.28% for the original and modified ModuLeaf, respectively – indicating a better overall fit for the modified ModuLeaf.

**Table 3:** Mean square error between Pinnacle model and measured profile tails.

*Depth*	*Field Size*	*Original Error, %*		*Modified Error, %*	
*cm*	cm×cm	*X*	*Y*	*X*	*Y*
5	1×1	0.04	0.30	0.03	0.15
	2×2	0.04	0.29	0.02	0.03
	3×3	0.11	0.30	0.02	0.02
	7×7	0.19	1.06	0.33	0.33
	10×10	0.75	2.99	0.57	0.36
10	1×1	0.64	0.96	0.33	0.19
	2×2	0.16	0.36	0.08	0.08
	3×3	0.13	0.26	0.09	0.08
	7×7	0.41	1.25	0.62	0.56
	10×10	1.50	4.06	0.97	0.74
	Average	0.40	1.18	0.31	0.25

While we are able to use the modified ModuLeaf in our clinic, the modifications are not available from the manufacturer. The addition of lead is relatively straightforward with the help of the manufacturer's field service engineer. However, ModuLeaf treatment deliveries require two plan files. The first plan file contains the ModuLeaf shapes from the treatment planning system. This is imported into Cosmic, the ModuLeaf controller application. Cosmic then exports a second file that contains the jaw positions and block codes for the linac. This second file is imported into the record and verify (R&V) system that is used with the linac.^(12)^ This R&V file must be modified so that instead of using jaws, the linac is given MLC leaf positions. This allows the user to specify that the MLC leaves behind the Y jaws should be closed. We have written in‐house software to create this R&V file. In the absence of such software, users will have to modify manually all this data in their R&V system. This is impractical, especially for IMRT treatments. We have informed the manufacturer of our modifications and they have entered the information into their databases for possible future improvements to the product.

## IV. CONCLUSIONS

The Pinnacle planning system model for the modified ModuLeaf is more accurate than the model for the original ModuLeaf, with a three‐fold reduction in leakage along the y‐axis (from 1.53% to 0.43%). While these modifications restrict our maximum treatment field size to 10 cm×10 cm, they also reduce the overall maximum leakage from 3.76% to 1.67%. However, this requires the use of in‐house software to close the linac's MLC leaves behind the Y jaws. While it also requires a slightly different clinical workflow, it provides leakage reduction, reduces the differences between the x‐ and y‐axis leakage distributions, and yields an improved beam model for treatment planning.
